# Supplementary material for: DMRforPairs: identifying Differentially Methylated Regions between unique samples using array based methylation profiles
Source: BMC Bioinformatics. 2014 May 15;15:141. doi: 10.1186/1471-2105-15-141 (PMC4046028; doi:10.1186/1471-2105-15-141)
Supplement: Additional file 2 — DMRforPairs output for the comparison of A431-MCF7 and NA17018-NA17105. Please start from the HTML files in each folder. Available via the BMC Bioinformatics website. [file 1471-2105-15-141-S2.zip › 1394847754114233_MOESM2_ESM/A431_MCF7/figures/7769.pdf]

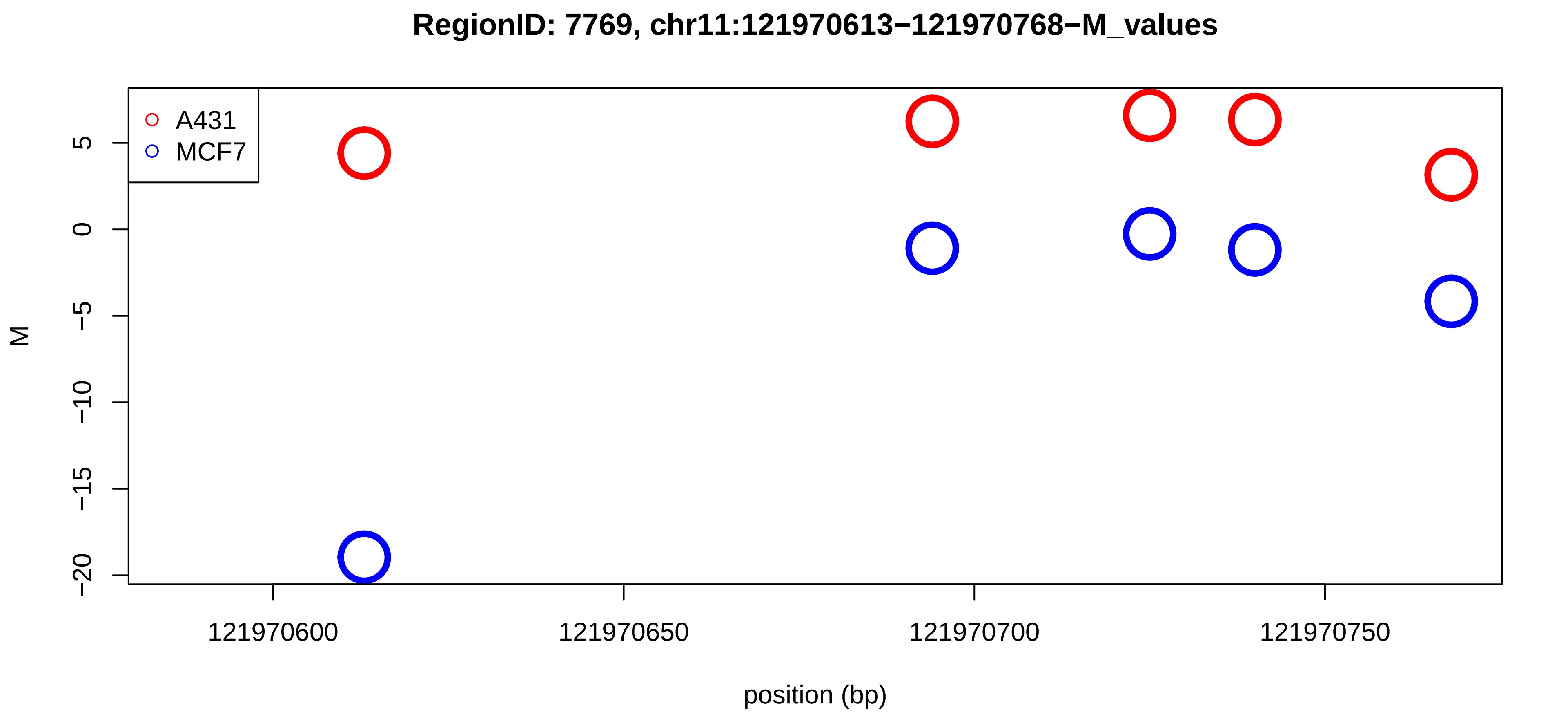
RegionID: 7769, chr11:121970613−121970768−M_values
A431
MCF7
M
5
0
−5
−10
−15
−20
121970600
121970650
121970700
121970750
position (bp)

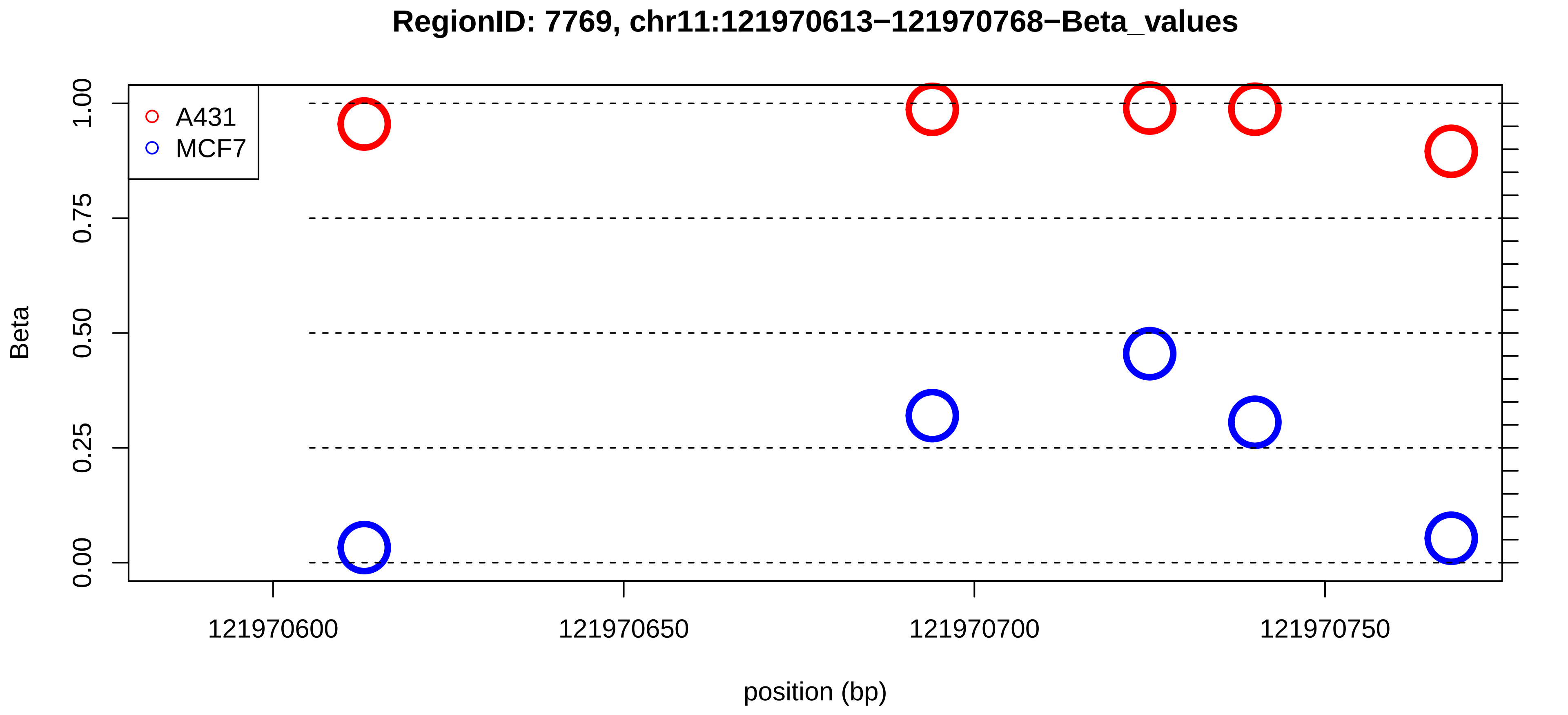
RegionID: 7769, chr11:121970613−121970768−Beta_values
A431
MCF7
Beta
1.00
0.75
0.50
0.25
0.00
121970600
121970650
121970700
121970750
position (bp)

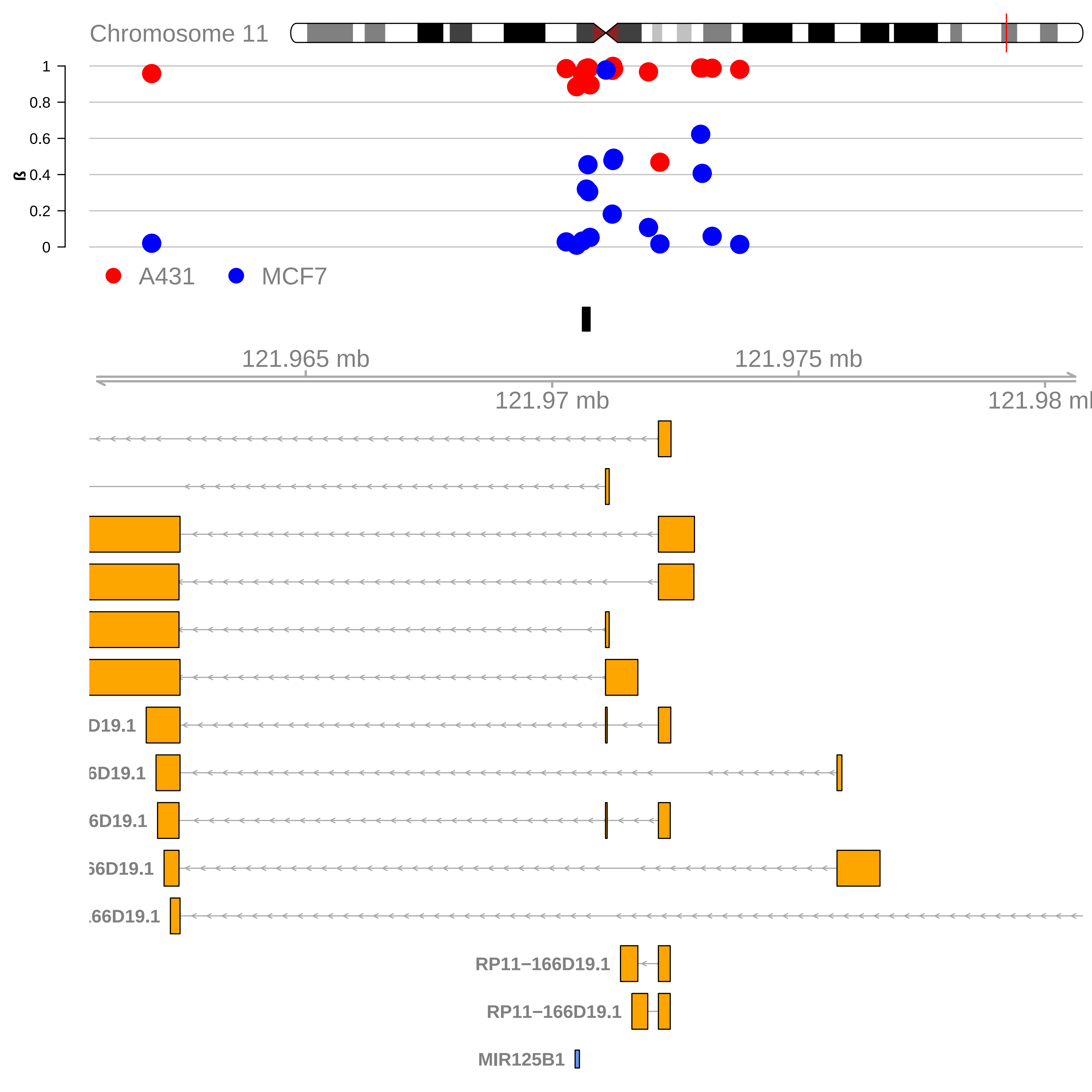

Chromosome 11
1
0.8
0.6
ß 0.4
0.2
0
A431
MCF7
121.965 mb
121.975 mb
121.97 mb
121.98 mb
D19.1
6D19.1
6D19.1
56D19.1
.66D19.1
RP11−166D19.1
RP11−166D19.1
MIR125B1
